# Effects of the number of drugs used on the prevalence of adverse drug reactions in children

**DOI:** 10.1038/s41598-020-78358-3

**Published:** 2020-12-07

**Authors:** Mayuko Sugioka, Tomoya Tachi, Takashi Mizui, Aisa Koyama, Azusa Murayama, Hayato Katsuno, Takuya Matsuyama, Satoshi Aoyama, Tomohiro Osawa, Yoshihiro Noguchi, Masahiro Yasuda, Chitoshi Goto, Hitomi Teramachi

**Affiliations:** 1 Laboratory of Clinical Pharmacy, Gifu Pharmaceutical University, 1-25-4, Daigakunishi, Gifu-shi, Gifu, 501-1196 Japan; 2 Department of Pharmacy, Gifu Municipal Hospital, 7-1, Kashima-cho, Gifu, Japan; 3 Laboratory of Community Health Pharmacy, Gifu Pharmaceutical University, Gifu, Japan

**Keywords:** Health care, Risk factors, Signs and symptoms

## Abstract

In pediatric individuals, polypharmacy would increase the prevalence of adverse drug reactions (ADRs). However, there is no report on the ADR increase adjusted for the influence of concomitant disease types. We conducted a retrospective study in pediatric patients to determine whether polypharmacy is a risk factor for ADR development, after the adjustment. Patients aged 1–14 years on medication who visited Gifu Municipal Hospital (Gifu, Japan) were included. We evaluated patient characteristics, ADR causality, ADR classification and severity, and ADR-causing drugs. We examined the association between ADR prevalence and number of drugs used. We performed multiple logistic regression analyses to investigate risk factors for ADR development. Of 1330 patients, 3.5% sought medical attention for ADRs. ADR causality was most often assessed as “possible,” with gastrointestinal ADRs being the most common. Grade 1 ADRs were the most and antibiotics were the most common suspected ADR-inducing drug. The multiple logistic regression analysis showed that ≥ 2 or ≥ 4 drug use, neoplasms, mental and behavioral disorders, and circulatory system diseases significantly increased ADR prevalence. Polypharmacy increased the prevalence of ADR resulting in hospital visits in children, after adjusting for the influence of disease types. Therefore, proactive polypharmacy control measures are necessary for children.

## Introduction

Adverse drug reactions (ADRs) are a major cause of outpatient visits and hospitalizations^1–3^. They account for 0.4–7.8% of all outpatient visits^1,2^, and 0.5%–37.5% of all inpatient hospitalizations^1,3^. Because the pharmacokinetics in children is different from that in adults^4^, the type and frequency of ADRs are specific to pediatric patients^5^. Several studies outside Japan have reported that the rate of outpatient visits and hospitalizations owing to ADRs in children is 0–11.0% and 0.41–10.3%, respectively^1,6^. However, there is a lack of similar studies in Japan. Analyzing and clarifying the ADRs resulting in hospital visits in children are important for appropriate medications in children.

Polypharmacy is commonly defined as the use of five or more prescribed medications for adults^7^. Polypharmacy in adults has been reported to be associated with an increased risk of ADRs, drug–drug interactions, hospitalizations, poor medication adherence, and mortality^8–10^. In contrast, polypharmacy has been reported as the use of two or more prescribed medications for children recently^11^. Although the increased risks may be extrapolated to children, most reports on the increased risks are limited to polypharmacy involving psychotropic medications or for psychiatric patients^11–15^. There are a few studies on the effects of polypharmacy on ADR prevalence in children with various disease types^16,17^. Using univariate analysis, one study conducted in Saudi Arabia demonstrated that the prevalence of ADRs in children (≤ 15 years of age) taking five or more drugs in a hospitalized setting was significantly higher than that in children taking less than five drugs^16^. Another study conducted in Germany demonstrated that, according to a multivariate analysis, the prevalence of ADRs in children (≤ 17 years of age) taking four or more drugs in non-institutionalized settings was significantly higher than that in children taking one drug^17^. However, these studies considered the effect of the health status, but not the effect of the disease types of the enrolled pediatric patients. Thus, although there are reports on the association of polypharmacy with ADRs in children, there is no report of analyses adjusted for the influence of concomitant disease types as confounding factors. Certain complicated diseases with accompanying conditions may influence the development of ADRs. For example, more ADRs are expected to occur in patients with psychiatric or neurological diseases and cancer compared with others^11,12^. Therefore, it is necessary to evaluate the influence of pediatric polypharmacy on ADRs, after adjusting for the influence of various types of diseases. Information on the effects of pediatric polypharmacy on ADRs is essential for clinical practice because it is expected that a reduction in the incidence of ADRs will reduce the burden on patients, and thus reduce the medical cost. Moreover, the information is also important for advancing and guiding research on clinical decision-making processes and for the formulation of clinical practice guidelines^11^.

The objectives of this study were to evaluate the prevalence of ADRs in pediatric outpatients and hospitalized patients and determine whether pediatric polypharmacy is a risk factor for hospital visits and hospitalizations owing to ADRs. Therefore, we conducted a retrospective study on the association between polypharmacy and the prevalence of ADRs in pediatric patients who visited a hospital or were hospitalized.

## Methods

### Study subjects

Eligible patients were children aged between 1 and 14 years who visited Gifu Municipal Hospital (Gifu, Japan) as unscheduled outpatients or were hospitalized for an emergency and taking one or more medications for one or more days at the time of visit or hospitalization between July 1, 2015 and December 31, 2015. The pediatric population in Japan is defined as children aged less than 15 years, and the drug usage in Japan are mostly determined by the cutoff age of 15 years. In this study, neonates and infants under one year including premature babies were not included because the ADRs may be influenced by breast milk containing drugs taken by their mothers and they cannot communicate ADRs to their mothers or doctors. Medication period at the time of visit or hospitalization was set to one or more days, as seen in many studies on pediatric polypharmacy^11^. The exclusion criteria were patients receiving a clinical trial.

Gifu Municipal Hospital is a secondary medical institution in Gifu Prefecture in Japan, and the Gifu City Holiday Emergency Center and the Pediatric Night Emergency Center are located within the hospital. Therefore, it is a complex hospital that provides primary and secondary health care.

### Details of the survey

The survey was conducted retrospectively using electronic medical records. The survey parameters were sex; age; diseases; drugs used at the time of hospital visit or hospitalizations (excluding topical drugs for local action); ADR prevalence and severity; clinical laboratory values; and medical records of physicians, pharmacists, and nurses. Diseases were classified using the International Statistical Classification of Diseases and Related Health Problems 10th Revision^18^. These survey parameters were investigated by manually checking and reading electric medical records.

The outcome of this study was the prevalence of ADRs that were extracted by referring to the Global Trigger Tool for Measuring Adverse Events^19^. The classification and severity of ADRs were evaluated based on the data retrieved from the medical records and clinical laboratory values. The Common Terminology Criteria for Adverse Events (CTCAE) version 5.0, which is based on Medical Dictionary for Regulatory Activities Japanese Version (MedDRA/J)^20^, was used to evaluate the classification and severity of ADRs. The ADRs were classified by system organ class according to MedDRA/J. The causality of ADRs was categorized using the Causality Assessment System^21^ published by the Uppsala Monitoring Centre, the field name for the World Health Organization (WHO) Collaborating Centre for International Drug Monitoring, into three levels: “certain,” “probable/likely,” and “possible.” Suspected drugs related to ADRs were classified by the Anatomical Therapeutic Chemical (ATC) classification^22^. Two pharmacists with at least 10 years of clinical experience and, when necessary, a physician extracted details of ADRs; assessed their severity classification, causality, and suspected drugs; and followed them up until discharge.

The cut-off point for the number of drugs used against the prevalence of ADRs was determined by plotting a receiver operating characteristic (ROC) curve. We then compared the prevalence of ADRs when only one drug was used with that when 2–3, 4–5, or ≥ 6 drugs were used. Thereafter, two groups were created using the cut-off point.

We analyzed our data by univariate and multivariate analyses in the following two stratifications: (i) two or more drugs, generally reported as pediatric polypharmacy^11^, (vs. one drug) and (ii) four or more drugs, with the cut-off point of the number of drugs used obtained from ROC curves in this study, (vs. three or less drugs).

### Statistical analysis

Fisher's exact test was used to compare the prevalence of ADRs in patients between groups using different number of drugs. We stratified patients by sex, age, number of drugs used (≥ 2 or ≥ 4), and presence/absence of disease. We then performed a univariate analysis (Fisher's exact test) to examine the difference in the proportion of patients affected by ADRs. We performed a multiple logistic regression analysis with the prevalence of ADRs at the time of hospital visits or hospitalizations as the dependent variable, and parameters with *P* < 0.25 in the univariate analysis as the independent variable. A *P* value of < 0.05 indicated a significant difference. We used IBM SPSS Statistics 24.0 J (IBM Corp., Armonk, New York) for statistical analysis.

### Ethics declarations

Based on the Ethical Guidelines for Medical and Health Research Involving Human Subjects (Ministry of Health, Labour and Welfare of Japan), written informed consent from patients was not required because this was a retrospective study of routinely collected data and did not require any interventions or interactions with patients. This study was approved by the ethics committees of Gifu Municipal Hospital (approval number: 349) and Gifu Pharmaceutical University (approval number: 28-8).

## Results

### Characteristics of the patients

The characteristics of the patients enrolled in this study are shown in Table 1. None of the patients fulfilled the exclusion criteria in this study. The total number of patients was 1,330, of which 760 were boys (57.1%). The median age was three years. The median number of drugs used was two, and diseases of the respiratory system were the most common in 78.9% of the patients.

**Table 1:** Characteristics of the patients.

	n = 1330
Sex (n (%))	
Boys	760 (57.1)
Girls	570 (42.9)
Age [years, median (25 percentile, 75 percentile)]	3 (2, 7)
3–6 years	515 (38.7)
7 years and older	335 (25.2)
Hospitalization	
Hospitalized	353 (26.5)
Not hospitalized (Outpatient visit)	977 (73.5)
Drugs used [n, median (25 percentile, 75 percentile)]	2 (1, 4)
Diseases (n (%))	
Certain infectious and parasitic diseases	394 (29.6)
Neoplasms	19 (1.4)
Diseases of the blood and blood-forming organs and certain disorders involving the immune mechanism	56 (4.2)
Endocrine, nutritional and metabolic diseases	200 (15.0)
Mental and behavioural disorders	19 (1.4)
Diseases of the nervous system	60 (4.5)
Diseases of the eye and adnexa	28 (2.1)
Diseases of the ear and mastoid process	72 (5.4)
Diseases of the circulatory system	26 (2.0)
Diseases of the respiratory system	1,049 (78.9)
Diseases of the digestive system	192 (14.4)
Diseases of the skin and subcutaneous tissue	208 (15.6)
Diseases of the musculoskeletal system and connective tissue	63 (4.7)
Diseases of the genitourinary system	38 (2.9)
Pregnancy, childbirth and the puerperium	1 (0.1)
Certain conditions originating in the perinatal period	7 (0.5)
Congenital malformations, deformations and chromosomal abnormalities	5 (0.4)
Symptoms, signs and abnormal clinical and laboratory findings, not elsewhere classified	247 (18.6)
Injury, poisoning and certain other consequences of external causes	125 (9.4)

Patients with two or more diseases were counted for each disease.

### Adverse drug reactions

Forty-six patients (3.5%) visited the hospital owing to ADRs. Of these patients, 34.8% were hospitalized (16/46), representing 4.5% (16/353) of pediatric emergency admissions. There were 54 ADRs that resulted in outpatient visits or hospitalizations with multiple ADRs in five of these patients. The results of the causality assessment of ADRs are shown in Table 2A. Most of the ADRs were classified as “possible” (66.7%), followed by “probable/likely” (22.2%) and “certain” (11.1%) causes. The results of ADR classification are shown in Table 2B. The most common diseases affecting specific body systems were “gastrointestinal disorders” (42.6%), and “skin and subcutaneous tissue disorders” (16.7%). The severity of ADRs is shown in Table 2C. Grade 1 ADRs were the most common (57.4%), followed by grade 2 (24.1%) and grade 3 (18.5%). The drugs that were most commonly associated with ADRs were “antiinfectives for systemic use” (55.6%), followed by “respiratory system” (18.5%) and “antineoplastic and immunomodulating agents” (11.1%) (Table 2D).

**Table 2:** Breakdown of adverse drug reactions.

	n = 54
	Number of events (%)
**A. Assessment of the causality of adverse drug reactions**	
Certain	6 (11.1)
Probable/ likely	12 (22.2)
Possible	36 (66.7)
**B. Classification of adverse drug reactions**	
Gastrointestinal disorders	23 (42.6)
Skin and subcutaneous tissue disorders	9 (16.7)
Investigations	6 (11.1)
Infections and infestations	4 (7.4)
General disorders and administration site conditions	3 (5.6)
Nervous system disorders	3 (5.6)
Metabolism and nutrition disorders	2 (3.7)
Blood and lymphatic system disorders	1 (1.9)
Immune system disorders	1 (1.9)
Psychiatric disorders	1 (1.9)
Respiratory, Thoracic and mediastinal disorders	1 (1.9)
C. Severity of adverse drug reactions*	
Grade 1	31 (57.4)
Grade 2	13 (24.1)
Grade 3	10 (18.5)
Grade 4	0 (0)
Grade 5	0 (0)
**D. Classification of suspected drugs related to adverse drug reactions**	
Antiinfectives for systemic use	30 (55.6)
Respiratory system	10 (18.5)
Antineoplastic and immunomodulating agents	8 (14.8)
Nervous system	4 (7.4)
Alimentary tract and metabolism	1 (1.9)
Blood and blood forming organs	1 (1.9)

Each adverse drug reaction was counted, even when there is more than one adverse drug reaction per patient.

*Grade refers to the severity of the adverse events according to Common Terminology Criteria for Adverse Events v5.0. Grade 1: Mild; asymptomatic or mild symptoms; clinical or diagnostic observations only; intervention not indicated. Grade 2: Moderate; minimal, local or noninvasive intervention indicated; limiting age appropriate instrumental activities of daily life. Grade 3: Severe or medically significant but not immediately life-threatening; hospitalization or prolongation of hospitalization indicated; disabling; limiting self care activities of daily life. Grade 4: Life-threatening consequences; urgent intervention indicated. Grade 5: Death related to AE.

### Relationship between the number of drugs used and the prevalence of drug reactions

The cut-off point obtained from the ROC curves (area under the curve, 0.630) was the use of four drugs. The prevalence of ADRs was significantly higher with the use of 4–5 or ≥ 6 drugs than with the use of one drug (Table 3). Therefore, we divided the analysis into two stratifications: use of two or more drugs, which is the most widely accepted definition for polypharmacy in children, and use of four or more drugs based on the cut-off point and the association between the number of drugs used and the prevalence of ADRs observed in this study.

**Table 3:** Prevalence of adverse drug reactions with number of drugs used.

Number of drugs used	Prevalence of adverse drug reactions	*P*
1	1.56% (7/448)	Control
2–3	3.60% (17/472)	0.063
4–5	4.48% (13/290)	0.021*
≥ 6	7.50% (9/120)	0.002*

Fisher's exact test. *P < 0.05.

### Univariate analysis

The results of the univariate analysis are shown in Table 4. The *P* value was less than 0.25 for the use of two or more drugs and four or more drugs, and six diseases including some infectious and parasitic diseases. It was more than 0.25 for sex and age. We did not include the following in the analysis owing to the small number of patients for each category: “diseases of the musculoskeletal system and connective tissue”; “pregnancy, childbirth, and the puerperium”; “certain conditions originating in the perinatal period”; and “congenital malformations, deformations, and chromosomal abnormalities.”

**Table 4:** Results of the univariate analysis.

	Prevalence of adverse drug reactions		*P*
	Yes (n = 46) n (%)	No (n = 1,284) n (%)	
**Patient characteristics**			
Sex (Boys)	26 (56.5)	760 (59.2)	0.931
Age (3 years and older)	32 (58.7)	818 (63.7)	0.416
Age (7 years and older)	11 (23.9)	324 (25.2)	0.976
Two or more drugs	39 (84.8)	843 (65.6)	0.004*
Four or more drugs	23 (50.0)	387 (30.1)	0.004*
**Diseases**			
Certain infectious and parasitic diseases	19 (41.3)	375 (29.2)	0.077
Neoplasms	4 (8.7)	15 (1.2)	0.003*
Diseases of the blood and blood-forming organs and certain disorders involving the immune mechanism	4 (8.7)	52 (4.0)	0.124
Endocrine, nutritional and metabolic diseases	11 (23.9)	189 (14.7)	0.087
Mental and behavioural disorders	4 (8.7)	15 (1.2)	0.003*
Diseases of the nervous system	2 (4.3)	58 (4.5)	0.656
Diseases of the eye and adnexa	1 (2.2)	27 (2.1)	0.631
Diseases of the ear and mastoid process	3 (6.5)	69 (5.4)	0.459
Diseases of the circulatory system	5 (10.9)	21 (1.6)	0.002*
Diseases of the respiratory system	35 (76.1)	1,014 (79.0)	0.638
Diseases of the digestive system	8 (17.3)	184 (14.3)	0.562
Diseases of the skin and subcutaneous tissue	5 (10.9)	203 (15.8)	0.365
Diseases of the musculoskeletal system and connective tissue	0 (0.0)	63 (4.9)	–
Diseases of the genitourinary system	1 (2.2)	37 (2.9)	0.618
Pregnancy, childbirth and the puerperium	0 (0.0)	1 (0.1)	–
Certain conditions originating in the perinatal period	0 (0.0)	7 (0.5)	–
Congenital malformations, deformations and chromosomal abnormalities	0 (0.0)	5 (0.4)	–
Symptoms, signs and abnormal clinical and laboratory findings, not elsewhere classified	8 (17.3)	239 (18.6)	0.834
Injury, poisoning and certain other consequences of external causes	5 (10.9)	120 (9.3)	0.438

Fisher's exact test. *P < 0.05.

### Multivariate analysis

A multivariate analysis was performed with the prevalence of ADRs as the dependent variable, and the use of two or more drugs and six diseases including some infectious and parasitic diseases, with a *P* value of < 0.25 in the univariate analysis, as independent variables. The results are shown in Table 5A. The prevalence of ADRs was significantly higher with the use of two or more drugs (OR, 2.86; 95% CI, 1.25–6.52; *P* = 0.012) and three diseases such as “neoplasms” (OR, 6.03; 95% CI, 1.42–25.62; *P* = 0.015), “mental and behavioural disorders” (OR, 5.68; 95% CI, 1.45–22.28; *P* = 0.013), and “disease of the circulatory system” (OR, 3.50; 95% CI, 1.04–11.76; *P* = 0.043).

**Table 5:** Multivariate analysis.

	Odds ratio (95% confidence interval)	*P*
**A. Use of ≥ 2 drugs**		
Two or more drugs	2.86 (1.25–6.52)	0.012*
Certain infectious and parasitic diseases	1.54 (0.83–2.87)	0.175
Neoplasms	6.03 (1.42–25.62)	0.015*
Diseases of the blood and blood-forming organs and certain disorders involving the immune mechanism	0.87 (0.22–3.43)	0.841
Endocrine, nutritional and metabolic diseases	1.29 (0.60–2.79)	0.513
Mental and behavioural disorders	5.68 (1.45–22.28)	0.013*
Diseases of the circulatory system	3.50 (1.04–11.8)	0.043*
**B. Use of ≥ 4 drugs**		
Four or more drugs	2.33 (1.27–4.28)	0.006*
Certain infectious and parasitic diseases	1.57 (0.84–2.92)	0.156
Neoplasms	5.37 (1.29–22.37)	0.021*
Diseases of the blood and blood-forming organs and certain disorders involving the immune mechanism	0.98 (0.25–3.86)	0.976
Endocrine, nutritional and metabolic diseases	1.30 (0.60–2.80)	0.508
Mental and behavioural disorders	5.10 (1.28–20.25)	0.021*
Diseases of the circulatory system	3.94 (1.18–13.14)	0.026*

Multiple logistic regression analysis. *P < 0.05.

The results of the multivariate analysis for the use of four or more drugs are shown in Table 5B. The prevalence of ADRs was significantly higher with the use of four or more drugs (OR, 2.33; 95% CI, 1.27–4.28; *P* = 0.006) and three diseases such as “neoplasms” (OR, 5.37; 95% CI, 1.29–22.37; *P* = 0.021), “mental and behavioural disorders” (OR, 5.10; 95% CI, 1.28–20.25; *P* = 0.021), and “disease of the circulatory system” (OR, 3.94; 95% CI, 1.18–13.14; *P* = 0.026).

There were eight parameters whose *P* value was < 0.25 in the univariate analysis, namely, the use of two or more drugs and four or more drugs; “certain infectious and parasitic diseases”; “neoplasms”; “diseases of the blood and blood-forming organs and certain disorders involving the immune mechanism”; “endocrine, nutritional, and metabolic diseases”; “mental and behavioural disorders”; and “diseases of the circulatory system.” Forty-six patients presented ADRs. In multiple logistic regression analysis, the number of outcome holders or non-holders, whichever is less, needs to be at least 10 times the number of independent variables used in the model^23^. However, the number of individuals who developed ADRs in this study was less than the number of independent variables multiplied by 10. Therefore, it was necessary to verify the robustness of our results. For this purpose, a multivariate analysis was performed with different numbers of independent variables by forward stepwise selection of variable with the lowest *P* value. The obtained *P* values and OR (Supplementary Table S1) were almost the same as those shown in Table 5, confirming the robustness of the multivariate analysis results.

## Discussion

We conducted a retrospective study to evaluate the prevalence of ADRs in children and to determine whether polypharmacy may be a risk factor for outpatient visits and hospitalizations owing to ADRs. According to our main findings, polypharmacy increased the prevalence of ADRs resulting in hospital visits in children, after adjusting for the influence of disease types, that is, it was clarified that polypharmacy was a risk factor for ADRs resulting in hospital visits.

The prevalence of ADRs was 3.5% in outpatients and 4.5% in hospitalized patients in the present study. This prevalence was consistent with those of a meta-analysis^1,6^. However, each study differs in the study design in many respects such as the target age, inclusion criteria, and study duration; thus, caution should be exercised when comparing the results of different studies.

The causality of ADRs was 11.1% for “certain,” 22.2% for “probable/likely,” and 66.7% for “possible.” In a previous report on pediatric patients in a hospital in the Netherlands, the proportions were 25%, 24%, and 51%, respectively, based on the Causality Assessment System of WHO^24^. In another previous report on pediatric patients in a hospital in India, the proportions were 0%, 30%, and 70%, respectively, based on the Causality Assessment System of WHO^25^. Therefore, the proportion of the causality of ADRs in this study was not inconsistent with the previous reports.

In the present study, the most common ADRs were “gastrointestinal disorders” followed by “skin and subcutaneous tissue disorders,” while in a previous study in the Netherlands, the most common ADRs were “gastrointestinal disorders” followed by “investigations” according to CTCAE classification^24^. Moreover, the most common ADRs affected “skin and appendages”, followed by “gastrointestinal” in previous studies conducted in Saudi Arabia^16^ and in India^25^. Therefore, the common ADRs in this study were not inconsistent with the previous reports although the classifications of ADRs in Saudi Arabia and in India were not similar with that of the present study. The ADRs of “gastrointestinal disorders” and “skin and subcutaneous tissue disorders” accounted for 59.3% of all ADRs in this study. “Gastrointestinal disorders” included diarrhea, constipation, and vomiting. “Skin and subcutaneous tissue disorders” included maculopapular rash. These ADRs are likely noticed by children themselves and are easily detected by their families. Consequently, these symptoms are easily communicated to medical professionals such as doctors and nurses, thereby contributing to the increased reporting of such events.

Serious ADRs of grade ≥ 3 severity accounted for 18.5% of all ADRs. Serious ADRs was 12% in CTCAE classification in the previous study conducted in the Netherlands^24^. A meta-analysis of pediatric patients hospitalized for ADRs reported a rate of 39.3% (95% CI, 30.4–47.9) for serious ADRs^26^, but it is difficult to make a strict comparison because this study included not only hospitalizations but also outpatient visits. It can be considered that the percentage in this study was not inconsistent with those reported previously.

“Antiinfectives for systemic use” comprised the highest percentage of drugs suspected to cause ADRs. This was followed by “respiratory system” and “antineoplastic and immunomodulating agents”. The suspected drugs were most commonly “cytostatics”, followed by “antibiotics” in the previous report from the Netherlands^24^. The suspected drugs were most commonly “anti-infective drugs” in the previous report from Saudi Arabia. The drugs prescribed by each hospital are different, and the frequency of use is also different. This may have led to the variation in the number of ADRs reported. Therefore, the common ADRs in this study were not inconsistent with the previous reports as a whole.

For drugs associated with a high number of ADRs, it is important to explain the possibility of ADRs to a pediatric patient and his/her parents at the time of drug administration and provide information about how to deal with the ADRs. It is also important to carefully monitor patients for ADRs of gastrointestinal disorders and skin and subcutaneous tissue disorders based on the type of ADRs (Table 2). Mild or moderate ADRs of grade ≤ 2 could be improved or prevented to a degree for which hospital visits or hospitalizations are not required. In addition, serious ADRs of grade ≥ 3 could also be improved to mild or moderate ADRs of grade ≤ 2 following these measures. Furthermore, it is essential for doctors to prescribe antibiotics more judiciously because, currently, more antibiotics than necessary are being prescribed for pediatric patients in Japan^27^.

A previous review discussed how many drugs should be considered polypharmacy in pediatric patients^11^. In the review, the most common comprehensive definition of pediatric polypharmacy was two or more concurrent medications for ≥ 1 day. It is important to clarify whether using two or more drugs increases the prevalence of ADRs or not. The rate of ADRs resulting from the use of 2–3 drugs was not significantly high compared with the use of one drug according to the results of Table 3. However, the results of the multivariate analysis showed that the use of two or more drugs also significantly increased the risk of outpatient visits and hospitalizations owing to ADRs. In particular, the results suggest that using four or more drugs influence the prevalence of ADRs.

The use of only one kind of drug is difficult in clinical practice, and the use of several kinds of drugs is often necessary. It is not realistic to establish the use of two or more drugs as the cut-off for avoiding polypharmacy-inducing ADRs. Furthermore, the criteria for the cut-off number of drugs that increase the ADRs would be necessary in clinical practice. The cut-off point obtained from the ROC curves was four drugs, and the prevalence of ADRs was significantly higher with the use of 4–5 or ≥ 6 drugs than with the use of one drug (Table 3). The number ≥ 4 drugs used could be the criteria for a cut-off number of drugs used that increases the ADRs in the clinical practice. The results of the multivariate analysis showed that the use of four or more drugs significantly increased the risk of outpatient visits and hospitalizations owing to ADRs, even after adjusting for the effect of the disease type. Polypharmacy (≥ 5 drugs used^16^ and ≥ 4 drugs used^17^) in pediatric populations has been reported to increase the prevalence of ADRs. However, no study has adjusted for the effects of disease types, as seen in this study. Herein, we could adjust for differences in the prevalence of ADRs owing to the disease type by incorporating disease as an independent variable in the multiple logistic regression analysis.

We would like to propose the use of four or more drugs as a possible cut-off number of drugs inducing ADRs based on the results of this study. In a previous report, four or more prescription medications were associated with clinical discrepancies^28^. Generally, more comorbidities tend to lead to the use of more drugs in pediatric patients. In these cases, four or more drugs may be required, along with those essential for treatment. From our findings in this study, minimizing the number of drugs administered through interventions based on careful benefit/risk analysis may help reduce the risk of ADRs in children.

Neoplasms, mental and behavioral disorders, and diseases of the circulatory system were identified as risk factors for outpatient visits and hospitalizations due to ADRs. Drugs used to treat neoplasms generally have a high prevalence of ADRs. The prevalence of ADRs is also known to be highly associated with medications for mental and behavioral disorders; at least one adverse drug event has been reported in all patients treated with haloperidol and chlorpromazine^29^. For patients with neoplasms or mental and behavioral disorders, careful attention to the signs of ADRs may prevent their development.

A major limitation of this study is its single-center retrospective design. In the future, it is necessary to conduct a prospective survey in multiple facilities and gather more evidence. Furthermore, we cannot rule out the possibility of bias in the reporting of medications used and ADRs by the patients, and in the description of medical records by healthcare providers. Furthermore, the number of drugs used in this study might be different from the number that patients actually took because patients did not communicate all their medications with healthcare providers. Although there was no significant difference in the prevalence of ADRs in different age groups (3 years and older, or 7 years and older) in this study, further research on the association between polypharmacy and ADRs in different age groups will be required.

In this study, we evaluated the prevalence of ADRs in pediatric patients and revealed that polypharmacy in children is a risk factor for ADR development in outpatients and hospitalized patients. Similar to that in older individuals, ADRs can be reduced in children by careful benefit/risk analysis and by eliminating polypharmacy through interventions such as prescription drug modification.

## Supplementary information

Supplementary Information.

## Data Availability

The datasets generated during and/or analyzed during the current study are available from the corresponding author on reasonable request.
